# Scientometric Analysis of Safety Sign Research: 1990–2019

**DOI:** 10.3390/ijerph18010273

**Published:** 2021-01-01

**Authors:** Jingqi Gao, Xiang Wu, Xiaowei Luo, Shukai Guan

**Affiliations:** 1 Department of Safety Science and Engineering, School of Engineering and Technology, China University of Geosciences (Beijing), Beijing 100083, China; gjq0421@cugb.edu.cn; 2 College of Engineering, City University of Hong Kong, Hong Kong, China; xiaowluo@cityu.edu.hk; 3 School of Reliability and System Engineering, Beihang University, Beijing 100191, China; gskonelight@126.com

**Keywords:** safety sign, scientometric, mapping knowledge domains, CiteSpace

## Abstract

The purpose of this paper is to summarize the research themes and hotspots of safety signs research between 1990 and 2019 through the scientometric analysis method. In total, 3102 articles of literature from the Web of Science core database were analyzed by the CiteSpace visualization tool and the results were displayed in mapping knowledge domains. The overall characteristics analysis showed that safety sign is an emerging research field in a rapid development stage—81.4% of the literature works were published in the past ten years, and the United States was in the leading position, followed by China and Canada. The keyword co-occurrence analysis indicated that traffic signs and driving safety were the most popular research topics and have been combined with simulation technology in recent years, whereby individual mental health has been added as an influential factor. The journals and category co-citation analysis showed that the safety signs research involved many subjects, mainly engineering, transportation and public safety. The results indicated that the safety signs research is multi-disciplinary, and it will continue to develop in various scientific domains in the future. The conclusions can provide help and reference for potential readers, as well as help with the sustainable development of safety signs research.

## 1. Introduction

Safety signs have the function of warning danger, avoiding or reducing accidents and decreasing personal injury, and its ultimate purpose is to promote safety-appropriate behavior [1]. Nowadays, safety signs are widely used in the world as a significant means of safety management. The document ISO3864-3 (2012) issued by the International Organization for Standardization defined safety signs as signs used to express specific safety information that composed of graphic symbols, safety colors, geometric shapes or borders and words, and it defined its understanding nature as “the meaning of signs of users interpretation” [2]. Laughery defined the notion of safety signs as a label, a statement or a picture in a manual, a posted sign, or an auditory alarm [3]. Therefore, all signs and labels which have safety issues related to people are regarded as safety signs in this paper, including traffic signs, a printed statement on a container box and so on.

There had been a significant increase in research on warnings since the middle 1980s [3]; later, scholars carried out many studies on it, such as safety signs’ effectiveness, design, identification, influence factors (e.g., individual characteristics) and other research topics [4,5,6,7,8,9,10,11]. Research on the summary review of safety signs has been continued in recent decades in order to summarize the research status and development prospects of safety signs. Miller and Lehto (1987) summarized the early literature [12], and Wogatler and Laughery reviewed safety signs and warnings during different development stages [3,11]. Despite those review papers analyzing the research traits of safety signs comprehensively, they are based on qualitative analysis instead of quantitative analysis. Therefore, in this paper, we decided to explore the research characteristics and development trend of safety signs quantitatively by the scientometric method.

Derek John de Solla Price was believed to be the pioneering figure of the scientometric analysis approach [13]. This method points out a new way of mass data statistical analysis for researchers. It explains how to find research frontiers uniquely, that is, to study the development trend of related fields by using the citation rules existing in various bodies of literature. The method is based on the co-author analysis, journal analysis, institution analysis, co-citation analysis, keyword analysis and other scientific methods, using visual analysis technology to explore the development trend, research hotspots and other traits of the subject. As a comprehensive review method, the scientometric method has been applied in engineering in recent years. Hosseini et al. applied it to analyze the characteristics of off-site construction [14]. Based on the bibliometric review and in-depth discussion, Jin et al. used VOSviewer software and the scientometric method to analyze the current situation and future research direction of construction safety [15]. Li et al. analyzed the safety science research structure by the mapping knowledge domains [16]. However, there is no research on safety signs based on the scientometric method.

Using scientometric to explore the safety signs research can help to improve the understanding of experts, scholars and the general public on the development traits, research status and significance of safety signs, and deepen researchers’ understanding of the internal development rules of it, so as to promote the sustainable development of safety signs research. Therefore, this paper tends to use the CiteSpace visualization tool designed by Dr Chen and his team [17] to conduct a scientometric analysis on the safety sign literatures from 1990 to 2019 included in the Web of Science Core Collection. Based on the analysis results, this paper will deduce and predict the future development direction of the safety sign, and explore the changing trend of the research focus over thirty years, so as to provide the overall situation of safety signs research in a systematic and macroscopic way for researchers, and put forward constructive suggestions for the future development of safety signs, and promote the safety sign science development. The main objectives of this study are as follows: (1) to analyze the research status of safety signs; (2) to analyze the distribution and cooperation of safety signs researchers and institutions; (3) to explore the research theme and hotspots of safety signs.

## 2. Materials and Methods

### 2.1. Research Tool

CiteSpace is selected as the visual analysis tool in this paper. This software was developed by Chaomei Chen of Drexel University and the WISE Lab of Dalian University of Technology [18]. Based on the citation analysis theory, Dr Chen expanded the application scope of author co-citation analysis and developed the Information Visualization CiteSpace software with Java, which can be used to explore the key path and knowledge turning points of a discipline domain evolution. Through a series of visual knowledge maps generated, CiteSpace explores the research status, research hotspots, evolution process and discipline structure of a scientific field, so as to grasp the research direction of institutions and authors and judge the classic literature and related assisted research based on preceding analysis. In international scientific research, CiteSpace software is widely used in more than sixty fields, including computer science, information science and medicine. Therefore, this paper uses CiteSpace 5.6, which is relatively stable and new at present, as the data analysis research tool, and combines with the bibliometric review method to conduct a scientometric analysis in the field of safety signs.

CiteSpace provides eleven kinds of function selection for the co-operation network and co-occurrence maps of citation literatures and the co-citation maps of the cited reference. The generated mapping knowledge domains can be used to explain the development status and changes of scientific structure. To facilitate readers to understand, the main structure of the science map is explained as follows. The map includes nodes, labels and lines. The node represents the object to be analyzed, and the more the object appears or is cited, the larger the node is. The color and thickness of the node’s inner ring indicate the occurrence frequency of the object in different time periods. The connection lines between nodes represent the co-occurrence or co-citation relationship; the lines’ thickness indicates the strength of the relationship, and the color corresponds to the first co-occurrence or co-citation time of nodes. Colors from cool to warm represent early to recent.

### 2.2. Data Sources

At present, the literature databases commonly used by scholars include Google Scholar, Scopus, PubMed and Web of Science. Among them, Web of Science (WoS) is a large-scale multidisciplinary core journal citation database, covering engineering, natural sciences, social sciences and other fields, and includes many of the world’s authoritative, high-impact academic journals. Meanwhile, studies have shown that the WoS database presents a better knowledge map effect when CiteSpace is used for visual analysis [19,20]. Therefore, for effectively analyzing the status quo and development trend of safety signs, this paper chose Web of Science as the sample database.

The sampling process is described below. First of all, the Web of Science Core Collection database was selected for basic retrieval. The search period was fixed between 1990 and 2019. The topic search could gain a more robust dataset than keyword search when investigating a rapidly growing field. Meanwhile, when using “safety sign”, “safety label”, and “warning” as search words, the literature that was found more closely fit demands using topic search instead of keyword search. Therefore, this paper used topic search and the word “safety” was used to limit the scope of the research works being found. Like the Chen et al. paper [21], our document types were set as “Article” and “Review”. A total of 3150 records were obtained from the WoS database. The records’ titles, abstracts and keywords were studied in detail before being taken as research samples. If the content of an abstract was related to safety signs and individual safety, such as “the FLR warning system has a potential to reduce the probability of grade crossing collisions” [22], thus the record was retained. However, some articles had the term related to safety signs or warnings in their abstracts, but focused on other objects, for example, Wu et al.’s research on carbon monoxide sources [23]. Those records were excluded. After filtering out less relevant records, the duplicate data samples were removed in CiteSpace and 3102 safety sign published literature records were retained as research samples in this paper.

## 3. Results

This section describes the analysis results of 3102 literature works covering thirty years from 1990 to 2019.

### 3.1. General Feature of Published Research

**Figure 1:**
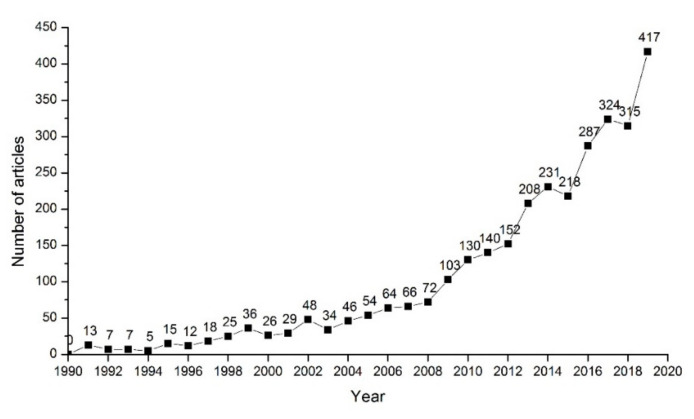
Annual trend of safety sign literature.

The overall characteristics of the number of published papers can reflect the direct results and development trend of safety sign research. The publication trend of safety signs research is shown in Figure 1.

Figure 1 shows that the research on safety signs was in its infancy in the 1990s, and its development speed was slow (138 published articles); from 2000 to 2008, 439 literatures were published; 81.4% of the safety signs articles were published between 2009 and 2019, and the research on it entered a period of rapid development. In addition, the cumulative number of published papers follows a J-like growth curve indicating that the growth rate of the number of safety sign publications is continuing to rise. As an eternal research topic in the field of safety science and engineering, safety signs research is expected to have more publications in the future.

**Figure 2:**
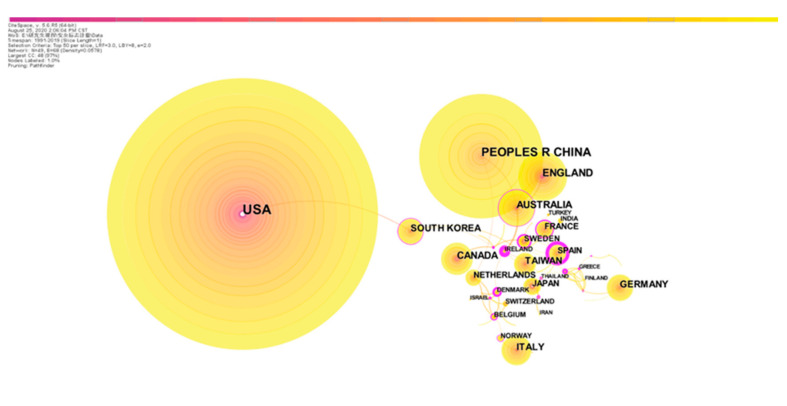
Countries or regions of safety signs research network.

The national or regional network map of safety signs research is shown in Figure 2. It showed that 36.91% of the published papers were from the United States. The following high-publication countries and regions are China (531 articles, 17.1%, excluding Taiwan), England (230 articles, 7.41%), Australia (171, 5.51%), Canada (149, 4.80%), Italy (145, 4.67%), Germany (135, 4.35%), South Korea (123, 3.97%), Japan (89, 2.87%) and the Netherlands (85, 2.74%). The network also indicated that safety signs research started early in the United States, England, Belgium and Australia. In comparison, South Korea, Netherlands and China are emerging countries in this research field. Moreover, about ninety countries and regions have published relevant articles from 1990 to 2019, and Figure 2 shows that both developed and developing areas were conducting research on safety signs. It indicates that safety signs research has already attracted the attention of all countries and regions, and it has become a research hotspot and key issue around the world. However, except for the lines among the UK, Australia and Ireland, other connections between nodes in Figure 2 are sparse, which means the cooperation between countries and regions is not close enough.

**Figure 3:**
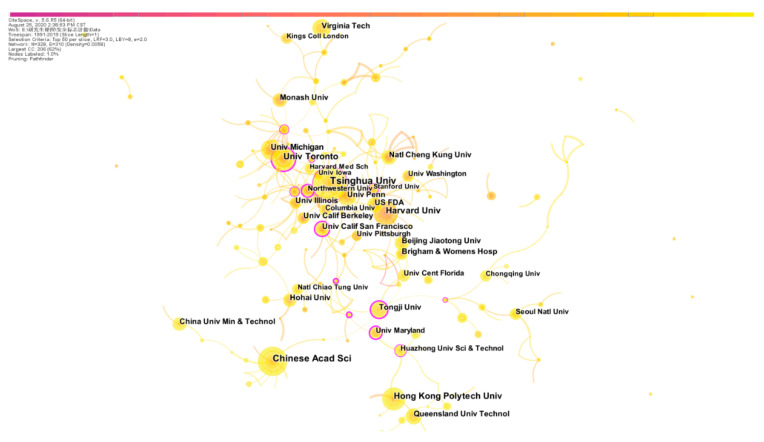
Institutions of safety signs research network.

Additionally, the institutions’ cooperation was analyzed, and the network is shown in Figure 3. The colors of lines between institutions in the network shows that institutions’ cooperation has happened more frequently in recent years, but it is not close enough. Moreover, data statistics show that Tsinghua University (35 published literatures), University of Chinese Academy of Sciences (32), Harvard University (27), University of Toronto (26) and the Hong Kong Polytechnic University (25) were the top five institutions. Chinese research establishments accounted for three in the number of published works. Moreover, the centrality of the node known as node importance can be understood as an article’s impact factor. The results of centrality showed that the top seven research institutions of thesis impact were University of California, San Francisco (0.33), University of Maryland (0.31), University of Southern California (0.29), Tongji University (0.28), University of Florida (0.28), University of Toronto (0.22) and Northwestern University (0.21), of which five were American universities and other two were, respectively, from China and Canada. This showed that the United States was in an authoritative position in the field of safety signs research, and both China’s and Canada’s research in this field also had high impacts.

To sum up, the current research on safety signs is still in the stage of rapid development. The research in this field was carried out earlier in North America and Europe, and relatively late in Asia and other areas. In terms of the published articles number and the paper impact, the United States is in an absolute leading position, and European countries are also at a high level. Asian countries or regions are at the forefront in the number of articles, however, except for China, others have an overall low paper impact. The above results indicate that the development of safety signs research is unbalanced. In addition to the influence of objective factors such as the situation of national development, the lack of close cooperation among international research institutions also affects the development of safety signs research to a certain extent.

### 3.2. Author’s Analysis

The co-author network map was generated in CiteSpace, and the node information was viewed by “Node Details”. The cooperation relationship and research field of the authors with a number of papers published more than or equal to four were summarized. The results are shown in Table 1.

**Figure 4:**
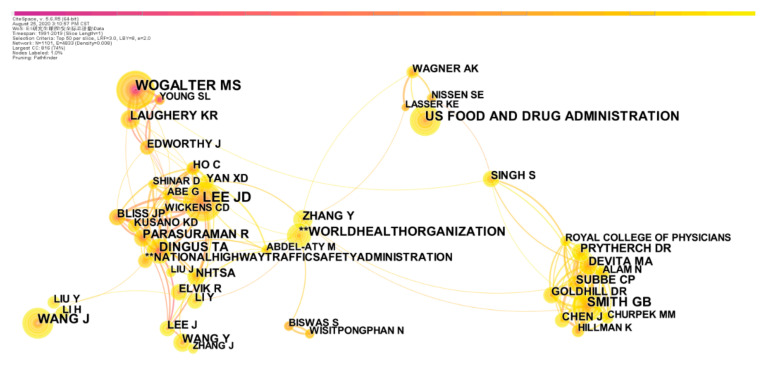
Author co-citation network of safety signs research.

To further determine the influential scholars in the field of safety signs and obtain the distribution of high cited authors, the authors co-citation analysis was carried out. Co-citation analysis refers to the co-citation relationship between two literature works when they appear together in the reference list of the third citation literature. A total of 1101 nodes and 4833 connections were obtained from the network map, which indicated that the authors cooperated closely. The author co-citation network is shown in Figure 4, where nodes with citation counts less than twenty are not displayed.

The results of Figure 4 show that the Wogalter and Laughery et al., Lee and Dingus et al., Smith and Devita et al. and Wang et al. were highly cited scholars in the field of safety signs. Wogalter, Smith and Wang also appeared in Table 1. Wogalter has studied the effectiveness and understandability of safety signs and published several studies with Laughery. Smith focuses on early warning systems in medical applications [24]. Wang has great influence in driving safety; he and his co-authors have investigated vehicle collision problems [25]. The research of Lee also focuses on driving safety and has great impact factors. His studies, such as [26], have been cited many times by scholars studying in traffic signs. The above four scholars can be regarded as authoritative researchers of safety signs. In addition, the results also show that the US Food and Drug Administration has played a significate role in safety signs research.

### 3.3. Journal Source and Category Analysis

**Figure 5:**
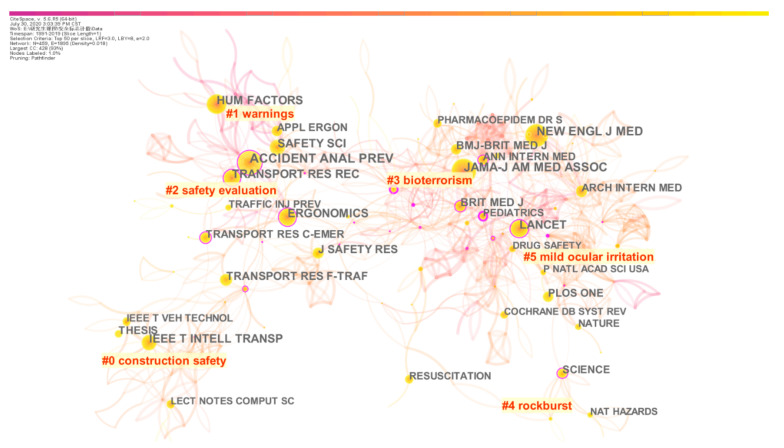
Journals co-citation clustering network of safety signs research. Note: Clusters are referred to in terms of the labels selected by the log-likelihood ratio test method (LLR).

Journal co-citation analysis can provide the distribution of important knowledge sources of safety signs. The network map was pruned, and we conducted a cluster analysis. Fifteen clusters were obtained; the largest six clusters are displayed in Figure 5.

According to the number of articles published in journals, Accident Analysis & Prevention (521), New England Journal of Medicine (410), Journal of the American Medical Association (407), Human Factors (311), Transportation Research Record (310), Lancet (307), Safety Science (304), IEEE Transactions on intelligent Transportation systems (261), Ergonomic (248), Transport Research Part F-Traffic Psychology and Behaviour (237), Journal of Safety Research (210) ranked in the top 10. The data indicated that safety signs were widely studied in medicine, transportation, safety science and human factor engineering, and this conclusion is consistent with the research fields of main authors in Table 1. Figure 5 clusters show the main research fields of safety signs, namely, Cluster#0 (50) “construction safety”, Cluster#1 (46) “warnings”, Cluster#2 (42) “safety evaluation”, Cluster#3 (38) “bioterrorism” (mainly research on child death, infant death and ethnic differences), Cluster#4 (33) “rock burst”, Cluster#5 (32) “mild ocular irritation” (mainly research on the safety of drugs or supplies, such as hair dye).

**Figure 6:**
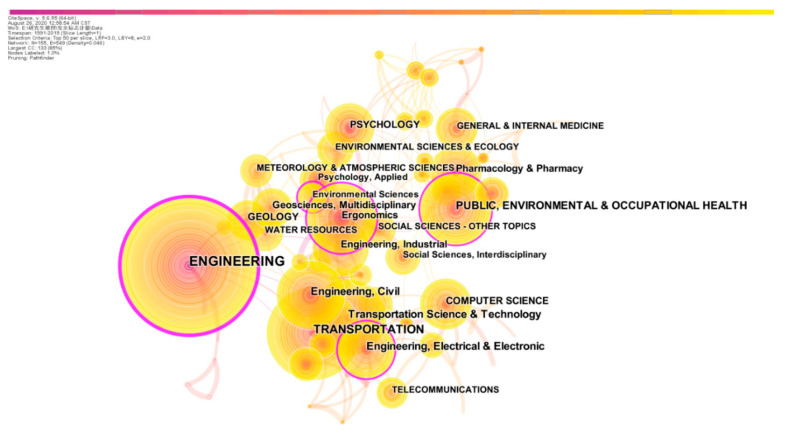
Category network of safety signs network.

A more intuitive structure of scientific field can be obtained by analyzing the category of safety signs. Only nodes whose citations are greater than or equal to 100 are displayed, and the co-occurrence network of each category is shown in Figure 6. The main scientific fields to safety signs are engineering (36.2%), transportation (17.4%), public, environmental and occupational health (11.3%), traffic science and technology (10.4%), civil engineering (9.2%), electrical and electronic engineering (8.3%), ergonomics (7.5%), computer science (7.1%), pharmacology and pharmacy (6.2%) and industrial engineering (5.6%).

### 3.4. The Research Theme of Safety Signs

Reference co-citation analysis is the most attractive function of CiteSpace. Through the co-citation analysis and clustering of safety signs, terms can be extracted to determine the research topics. Table 2 lists the clusters of co-citation references of ten safety signs clusters by size. The cluster’s silhouette can reflect the quality of it, that is, the more the silhouette value tends to 1, the higher the consistency of members in the cluster [21]. The ten clusters in the table below all have high homogeneity, especially Cluster#2 and #6, with a silhouette of 1.000. Each cluster label is marked by log-likelihood ratio (LLR).

Chen pointed out that the average year of a cluster can indicate its recency [21]. The results in Table 2 showed that the average year of the ten main clusters was between 2003 and 2014, and Cluster#3 “various safety-critical situation” was a recent research topic in the field of safety signs. Cluster#0 and #7 focus on a medical early warning system and related disease-risk safety warnings; Cluster#2, #5 and #9 are targeted research topics on safety sign identification under construction site conditions, traffic sign identification under heavy fog conditions and water safety signs, respectively; Cluster#4 and #6 aim at driving safety and safety signs under vibration mode analysis. Table 3 and Table 4 list the main cited references and citing articles of Cluster#1 and #3, to show the key research points.

Cluster#1 is named “advisory warning”. Kakkasageri reviewed the vehicle ad-hoc network in-road information recognition and other safety-related application [27], and Tak proposed surrogate safety measures as safety indicators of vehicle rear-end collision risk [28]; those two articles are the main citing articles in this cluster. Biswas (2006) is the most cited reference in Cluster#1 [29]. The title of Cluster#3 is “various safety-critical situation”, and the first two citing papers are Susan Winkler’s articles—the first article measured the drivers’ behaviors and the comprehensibility of warning signs by conducting a psychological driving simulator experiment, and the second one simulated multi-stage collision warning under different safety-critical situations [30,31]. The main references in Cluster#3 were mostly from the Accident Analysis and Prevention, and Jermakian was cited the most [32].

To summarize, the research frontier of safety signs mainly focuses on traffic and driving safety. The research more explores the problems of driving safety and traffic sign identification under extreme weather or other dangerous emergency conditions. In the ten major clusters, main references and citing articles in Cluster#1, #3, #4, #5, #6 and #9 are the studies on traffic signs and driving safety, with average years from 2003 to 2014, which shows that traffic and driving safety has always been an active research topic in the safety signs research. In addition, with the continuous progress of computer simulation and other technology, driving simulation experiment has become a cutting-edge topic in the field of traffic safety. Moreover, medicine is also a major research topic in safety signs, for example, Judy Edworthy has studied the safety signs of auditory warnings in medical institutions [33,34]. Additionally, the study of safety signs recognition at the building site and water warnings account for a larger proportion as well.

### 3.5. The Hotspots of Safety Signs Research

**Figure 7:**
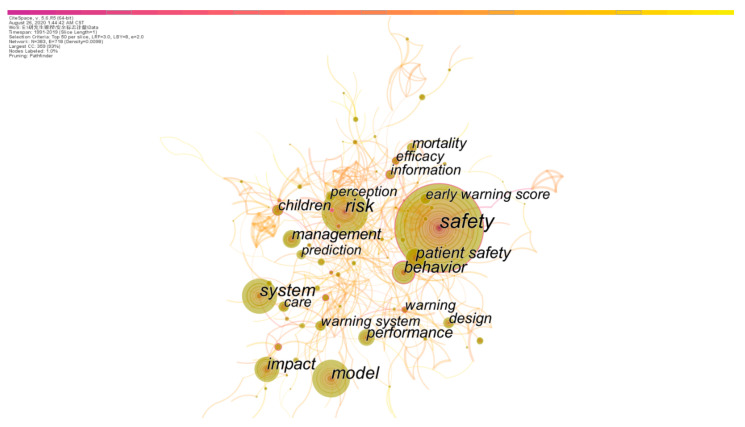
Keyword co-occurrence network.

The keywords can represent the core content of existing studies. Thus, the co-occurrence analysis on the keywords of literature from 1990 to 2019 was carried out to investigate the research hotspots of safety signs research. After pruning and clustering, the keyword co-occurrence network was obtained. Figure 7 shows the keyword node labels with more than and equal to fifty citations. Through co-occurrence analysis and the clustering operation of keywords, twenty-one clusters were obtained. Table 5 summarizes the largest five clusters and the most active cited articles.

It can be seen from Table 5 that prescription drug packaging labels, injury severity, driving collision, product safety signs, and method efficacy are the hot topics of safety signs research. In addition, the number of burst keywords in each cluster was summarized in the table. In CiteSpace, the more burst nodes of a cluster indicate that the cluster is more active and, to some extent, represents the emerging trends [35]. In the co-occurrence clustering of safety signs keywords, Cluster#1 “injury”, Cluster#0 “physician” and Cluster#3 “consumer product safety” have more burst keyword nodes, which means that the three clusters can represent the active research hotspots of safety signs around the average year of the clustering.

**Figure 8:**
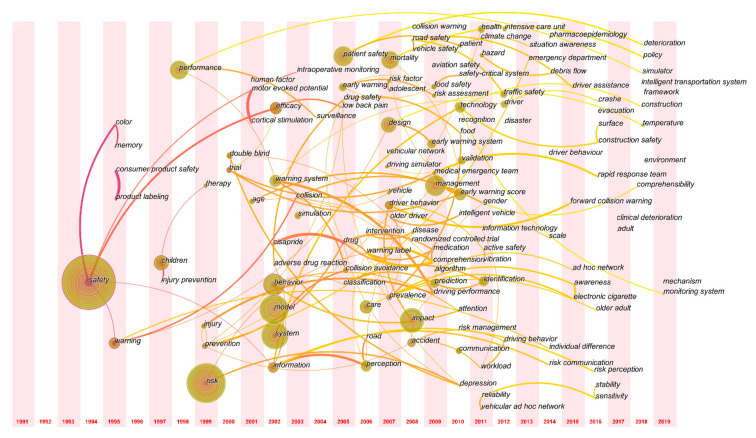
Time-zone view of keyword co-occurrence network of safety signs research.

To further analyze the research hotspots, the keyword co-occurrence network is displayed in the time-zone view (Figure 8). The position of nodes had been adjusted to the center of the year bar to obtain a clearer view of the temporal traits of safety signs research topics.

Figure 8 shows that the number of research topics on safety signs has increased since 2006. Looking at the whole figure, it is easy to notice that nouns related to driving safety and drug safety appear frequently. The keywords of safety signs research from 2016 to 2019 were observed carefully to obtain the recent research hotspots, which mainly include construction safety, collision warning system, comprehensibility, intelligent transportation system, deterioration, temperature, monitoring system, and simulator. Among them, “monitoring system” was mentioned many times in references co-citation analysis and clustering, indicating it is a relatively active research theme and an emerging research hotspot in 2019.

## 4. Discussion

### 4.1. Main Findings

This study explored 3102 safety signs literature works of the WoS core database from 1990 to 2019 using visualization technology, and systematically analyzed the overall characteristics, authors relationship, journal source, subject category structure of safety signs research through co-occurrence, co-citation and time-zone view analysis. This paper presents the first scientometric study on safety signs, aiming to investigate and explore the research theme and hotspots in this field, which provides a reference for the future research direction of safety signs, and also contributes to the scientific and sustainable development of safety signs research.

Based on the general features analysis of safety signs published research, safety signs research was in the stage of rapid development. The co-occurrence analysis results of countries or regions and research institutions showed that the United States had the earliest and most research on safety signs, and there was relatively more in European areas. China has carried out study on safety signs relatively late, but the number of published papers is in a leading position, and also it has a certain influence in this field. However, the cooperation between countries and regions is not close enough. It is mainly concentrated within a country or region. Meanwhile, the research affiliations also have the problem of imbalances. Most of the influential safety signs research institutions and scholars are in America. The number of publications, papers’ impact and important organizations indicates that the US is in the leading position of safety signs research. With the help of globalization, the cooperation of safety signs research is believed to be further develop.

Through the journal co-citation analysis and category co-occurrence analysis, this paper pointed out that engineering, transportation, public health and occupational safety, and human factors engineering were the main scientific domains of safety signs study. Journal co-citation clustering results showed that construction safety, products of health safety and environmental safety were the main research issues of safety signs. Chan et al. studied the comprehensibility of industrial safety signs in mainland China [9]. Smalley et al. studied the abuse and misuse of Cisapride and proposed that a clear indication of safety signs could reduce the drug safety risks [36]. Lasser [37] and Kurian [38] put forward similar viewpoints in drug research that the safety labels in prescription medications could reduce the dose of the drug. Sorensen pointed out that the establishment of warning system was of great significance to the early warning of weather disease in the article “Hazard Warning Systems: Review of 20 Years of Progress” [39]. The results showed that safety signs have been widely discussed in various fields.

This paper used the reference co-citation analysis, keyword co-occurrence analysis and keyword timeline view to explore the research topics and hotspots. The results showed that the research themes of safety signs included medical early warning, safety signs recognition of construction site and water site, traffic signs effectiveness and driving safety. The most major topic is traffic driving safety, which accounted for 60.0% of the ten largest clusters, and has been the main topic conducted over ten years. At present, with the development of computer technology, the research on driving safety is moving towards dynamic detection. Kakkasageri, Winkler and other main scholars used high-tech technology to investigate the driving safety and the effectiveness of traffic signs under various conditions (e.g., fog weather, automatic driving), while the keyword timeline network also illustrated that the “intelligent transportation system” and “monitoring system” are the emerging hotspots in 2018 and 2019. The keyword co-occurrence analysis showed that prescription medication packaging labels, product safety signs, driving collision and other issues were the hotspots of safety signs research. The burst inspection indicated that those researched hot topics were relatively active around 2003 and 2008. Moreover, Figure 8 showed that driving collision, safety signs comprehensibility and environmental safety have been the main hotspots in the last four years of safety signs research. Analysis on research themes, hotspots and scientific categories indicated that safety signs, as a major topic of safety science, is multi-disciplinary, which is the same as Paul et al.’s previous research conclusion [40]. The analysis of research themes and hotspots in the field of safety signs can provide help for scholars to better understand the research traits and developments rules of safety signs, provide reference basis for sign designers or relevant standard makers and provide a reference for future researchers to determine research topics. According to Figure 8 and literature review, the research topics of safety signs started from the 1990′s studies on safety signs’ color and performance [41,42] to the research on the recognitive and comprehensibility of safety signs by combing computer simulation and psychological experiments [1,43,44,45], as well as the investigation on individual safety problem, so as to achieve the fundamental purpose of safety signs, that is, reducing individual unsafe behavior. Those changes indicated that safety signs research is developing in multiple disciplines and showed the diversification of safety signs research. It can be predicted that the safety signs research will continue to develop in various scientific domains, and more scholars from different fields will pay attention to and promote further development of safety signs research, so as to improve the research system of safety signs.

### 4.2. Theoretical and Practical Implications

Safety signs, as an important safety measure for safety management in workplace, can mobilize individual’s psychological state to respond to objects or environments that threaten the individual’s health and safety. Therefore, exploring the safety signs research works by scientometric analysis would contribute to the current safety research and effectively promote the sustainable development of safety signs and safety management.

In addition, this paper indicated that traffic safety signs and driving safety are the hotspots and main research topics of safety signs, and using psychology experiments to investigate individual comprehensibility and cognition towards safety signs has also become a hot issue in recent years. It can provide research directions for potential readers, which will push the development of discipline so as to reduce individual risk-taking behaviors and ensure both personal safety and property safety in the workplace.

### 4.3. Limitations and Future Research Directions

Although this paper made a comprehensive exploration based on 3102 safety signs literature works, there are still some limitations. Firstly, this paper only considered the literatures written in English in the Web of Science core database, which may be limited. Additionally, this paper studied safety signs from multiple perspectives, including authors and journals, scientific domains structure, but the pertinence is somewhat weak.

In future analysis, we will focus on the research frontier of knowledge structure or a specific type of safety signs, e.g., prohibited signs, and expand the sample data to conduct a more in-depth discussion, thus to provide a more accurate reference for scholars and promote the sustainable development of safety signs research.

## 5. Conclusions

This study used CiteSpace 5.6 to analyze 3102 core literature works, written in English, by using the scientometric method and discussed the research status and development trend of safety signs. The main conclusions are as follows.

At present, safety signs are in a stage of rapid development, involving many scientific domains, such as engineering, medicine and transportation. As a worldwide research topic, the United States is in the leading position, China and Canada also have significant places in this field. The problem of less cooperation and imbalance development between countries and institutions still persists, but it may improve due to globalization. The main research topics and hot issues of safety signs are traffic signs and driving safety, and these have been the major research theme this past decade. With the help of simulation technology, the research on the driving signs recognition under different conditions is increasing and it will continue in the future. In addition, the safety signs of prescription medications and product packaging, the comprehensibility and cognition of safety signs are also a major theme of safety signs research. This paper predicts that safety signs will continue to develop in various disciplines, and gradually turn to the study of the simulation dynamic process to guarantee individual safety.

## Figures and Tables

**Table 1:** Cooperation and research interest of the main authors of safety signs.

Author Name	Number of Published Articles	Year ^1^	Co-Authors (Frequency)	Main Research Interests
Hampton C. Gabler	10	2014	Kristofer D. Kusano (5)	Traffic safety
M.S. Wogalter	9	1995	Kalsher, M.J.; N.C. Silver Laugher, Kenneth R. (2)	Significance and comprehensibility of safety signs
Xiaoyuan Wang	7	2018	Yaqi Liu; Fang Wang; Yuanyuan Xia (4)	Traffic safety
Jane Sandall	7	2012	Nicola Mackintosh (6)	Patient safety
Yina Wu	5	2018	Mohamed Abdelaty (5)	Traffic safety
Heng Li	5	2018	Dong Chao; Ding Lieyun; Luo Hanbin; Luo Xiaochun; Wong Arnold Yu Lok (2)	Construction safety
Gary B. Smith	5	2010	Peter Griffiths (3)	Patient safety
Faisal Khan	5	2014	Paltrineri, Nicola; Cozzani, Valerio (2)	Industrial safety (emergency)
Yiqi Zhang	4	2016	Changxu Wu (4)	Traffic safety (warnings)
Man Ho Kim	4	2010	Lee, Suk; Lee, Kyung Chang (3)	Traffic safety (intelligent vehicles)
Julie Considine	4	2016	Judy Currey (4)	Patient safety
Juancarlos Cano	4	2012	Francisco J. Martinez; Carlos T. Calafate (4)	Warnings
Jianqiang Wang	4	2015	KeqiangLi (2)	Driving safety

^1^ “Year” means the time when the author and co-author jointly published the first article in 1990–2019.

**Table 2:** Major clusters of co-cited references.

Cluster ID	Size	Silhouette	Label (LLR)	Year Ave.
0	125	0.838	maternal early warning criteria	2012
1	84	0.896	advisory warning	2009
2	52	1.000	construction site	2013
3	44	0.916	various safety-critical situation	2014
5	42	0.933	fog condition	2012
4	42	0.892	vibration pattern	2013
6	35	1.000	pattern analysis	2003
7	34	0.995	safety warning	2007
9	31	0.996	water-sport prohibitive symbol	2009

**Table 3:** Cited references and citing articles of Cluster #1.

Cluster #1 Advisory Warning			
Cited References		Citing Articles	
Cites	Author (Year) Journal, Volume, Page	Coverage %	Author (Year) Title
14	Biswas, S. (2006), IEEE COMMUN MAG, V44, P74	7	Kakkasageri, M.S. (2014) Information management in vehicular Ad Hoc networks: a review.
12	Wisitpongphan, N. (2007), IEEE WIREL COMMUN, V14, P84	7	Tak, Sehyun (2015) Development of a deceleration-based surrogate safety measure for rear-end collision risk.
8	Sengupta, R. (2007), J INTELL TRANSPORT S, V11, P143	6	Rhiu, Ilsun (2015) Research issues in smart vehicles and elderly drivers: a literature review.
7	Hartestein, H. (2008), IEEE COMMUN MAG, V46, P164	5	Song, Xiang (2015) Key parameters estimation and adaptive warning strategy for rear-end collision of vehicle.
6	Falk, B. (2009), TRANSPORT RES F-TRAF, V12, P1	5	Maag, Christian (2015) Car gestures—advisory warning using additional steering wheel angles.

**Table 4:** Cited references and citing articles of Cluster #3.

Cluster #3 Various Safety-Critical Situation			
Cited References		Citing Articles	
Cites	Author (Year) Journal, Volume, Page	Coverage %	Author (Year) Title
15	Jermakian, J.S. (2011), ACCIDENT ANAL PREV, V43, P732	20	Winkler, Susann (2018) How to warn drivers in various safety-critical situations—different strategies, different reactions.
12	Cicchino, J.B. (2017), ACCIDENT ANAL PREV, V99, P142	17	Winkler, Susann (2018) Practice makes better—learning effects of driving with a multi-stage collision warning.
9	Yan, X.D. (2015), TRANSPORT RES C-EMER, V51, P231	6	Kaplan, Sigal (2012) Associating crash avoidance maneuvers with driver attributes and accident characteristics: a mixed logit model approach.
9	Fildes, B. (2015), ACCIDENT ANAL PREV, V81, P24	6	Kaplan, Sigal (2012) The application of the random regret minimization model to drivers’ choice of crash avoidance maneuvers.
7	Reagan, I.J. (2016), TRAFFIC INJ PREV, V17, P827	6	Cicchino, Jessica B (2018) Effects of lane departure warning on police-reported crash rates.

**Table 5:** Keyword co-occurrence clusters’ names and main cited references.

Cluster ID	Size	Label (LLR)	Year Ave.	The Most Active Cited Reference (Author Title)	Number of Burst Keywords
#0	53	physician	2006	Davis, Terry C. (2009) Improving patient understanding of prescription drug label instructions.	8
#1	38	injury	2008	Wogalter, M.S. (1999) The relative contributions of injury severity and likelihood information on hazard-risk judgments and warning compliance.	9
#2	36	forward collision	2005	Cualain, D.O. (2012) Automotive standards-grade lane departure warning system.	6
#3	36	consumer product safety	2003	BRAUN, C.C. (1995) Interaction of signal word and color on warning labels—differences in perceived hazard and behavioral compliance.	8
#4	35	efficacy	2006	Cheng, Christine M. (2009) A review of three stand-alone topical thrombin for surgical hemostasis.	0

## Data Availability

The data presented in this study are available in the Web of Science core database.
